# Monitoring Changes in TMS-Evoked EEG and EMG Activity During 1 Hz rTMS of the Healthy Motor Cortex

**DOI:** 10.1523/ENEURO.0309-23.2024

**Published:** 2024-04-11

**Authors:** Stefan Schoisswohl, Carolina Kanig, Mirja Osnabruegge, Desmond Agboada, Berthold Langguth, Roman Rethwilm, Tobias Hebel, Mohamed A. Abdelnaim, Wolfgang Mack, Wolfgang Seiberl, Manuel Kuder, Martin Schecklmann

**Affiliations:** ^1^Department of Psychiatry and Psychotherapy, University of Regensburg, 93053 Regensburg, Germany; ^2^Department of Human Sciences, Institute of Psychology, Universität der Bundeswehr München, 85579 Neubiberg, Germany; ^3^Department of Human Sciences, Institute of Sport Science, Universität der Bundeswehr München, 85579 Neubiberg, Germany; ^4^Department of Electrical Engineering, Universität der Bundeswehr München, 85579 Neubiberg, Germany

**Keywords:** electroencephalography, motor-evoked potentials, neuroplasticity, repetitive transcranial magnetic stimulation, TMS-EEG, TMS-evoked EEG potentials

## Abstract

Repetitive transcranial magnetic stimulation (rTMS) is a non-invasive brain stimulation technique capable of inducing neuroplasticity as measured by changes in peripheral muscle electromyography (EMG) or electroencephalography (EEG) from pre-to-post stimulation. However, temporal courses of neuromodulation during ongoing rTMS are unclear. Monitoring cortical dynamics via TMS-evoked responses using EMG (motor-evoked potentials; MEPs) and EEG (transcranial-evoked potentials; TEPs) during rTMS might provide further essential insights into its mode of action – temporal course of potential modulations. The objective of this study was to first evaluate the validity of online rTMS-EEG and rTMS-EMG analyses, and second to scrutinize the temporal changes of TEPs and MEPs during rTMS. As rTMS is subject to high inter-individual effect variability, we aimed for single-subject analyses of EEG changes during rTMS. Ten healthy human participants were stimulated with 1,000 pulses of 1 Hz rTMS over the motor cortex, while EEG and EMG were recorded continuously. Validity of MEPs and TEPs measured during rTMS was assessed in sensor and source space. Electrophysiological changes during rTMS were evaluated with model fitting approaches on a group- and single-subject level. TEPs and MEPs appearance during rTMS was consistent with past findings of single pulse experiments. Heterogeneous temporal progressions, fluctuations or saturation effects of brain activity were observed during rTMS depending on the TEP component. Overall, global brain activity increased over the course of stimulation. Single-subject analysis revealed inter-individual temporal courses of global brain activity. The present findings are in favor of dose-response considerations and attempts in personalization of rTMS protocols.

## Significance Statement

Repetitive transcranial magnetic stimulation (rTMS) is a non-invasive brain stimulation method capable to induce neuroplasticity. Usually, changes in peripheral muscle or brain activity from pre-to-post stimulation serve as an indicator for TMS-related consequences. Monitoring temporal changes of these indicators during rTMS might provide essential information on how many pulses are necessary to induce modulation, respectively, change cortical excitability. In the present study, we show different temporal progressions, fluctuations, and effect plateaus of cortical excitability dependent on the indicator of interest. In general, global brain activity increased with the number of applied pulses. Further, inter-individual differences in the temporal course of global brain activity were observed. Our results suggest a consideration of dose-response dependencies and advocates approaches in customizing rTMS protocols.

## Introduction

Transcranial magnetic stimulation (TMS) represents a safe and well-tolerated (Lerner et al., 2019) non-invasive brain stimulation method in which transient high-intensity electromagnetic pulses are produced by a coil that is located over the scalp. The electromagnetic field traverses the cranial bone and depolarizes subjacent cortical neurons by inducing current flow (Barker et al., 1985; Lefaucheur, 2019). Single TMS pulses applied over the primary motor cortex typically evoke contralateral peripheral muscle responses as measured via electromyography (EMG). The amplitude of these motor evoked potentials (MEPs) is used as an indicator for cortical excitability (Rossini et al., 2015). The rhythmical application of single TMS pulses at certain frequencies is termed repetitive TMS (rTMS) and is capable of modulating cortical excitability outlasting the stimulation period as a function of the applied frequency (Fitzgerald et al., 2006; Ziemann et al., 2008). It is assumed that low frequencies induce cortical inhibition and high frequencies evoke cortical excitation via long-term depression (LTD), respectively, long-term potentiation (LTP) of synaptic strength (Hallett, 2000; Maeda et al., 2000; Fitzgerald et al., 2006; Chervyakov et al., 2015). Based on its ability to induce such neuroplastic changes rTMS is applied in a broad spectrum of fundamental and clinical research as well as in the treatment of several neuro-psychiatric conditions (Lefaucheur et al., 2020; Antal et al., 2022).

Recently electroencephalography (EEG) has been used in combination with TMS in order to measure direct cortical responses with high temporal resolution (Bergmann et al., 2016). It was shown, that single TMS pulses trigger evoked brain activity so-called TMS-evoked EEG potentials (TEPs) (Ilmoniemi et al., 1997), which provide a direct readout for cortical dynamics (Thut and Pascual-Leone, 2010). The typical TEP waveform after single TMS pulses applied to the primary motor cortex in healthy subjects is characterized by varying negative and positive peaks – e.g., N15, P30, N45, P60, N100 reflecting cortical TMS-related (re-)activity (Bonato et al., 2006; Lioumis et al., 2009; Ahn and Fröhlich, 2021). Accordingly, TEPs were used to evaluate the neuroplastic consequences of rTMS (Goldsworthy et al., 2021). It was demonstrated that rTMS is capable to modulate certain TEP components and that these could potentially serve as markers for neuroplastic changes (Thut and Pascual-Leone, 2010; Casula et al., 2014; Voineskos et al., 2021; Zhou et al., 2022). The majority of rTMS studies in combination with EEG investigated TEPs before and after rTMS (offline effects) (Tremblay et al., 2019). Monitoring and especially analyzing electrophysiological measures (TEPs, MEPs) while rTMS is applied (online effects), has the potential to provide further essential insights into the modes of action of this neuromodulation technique e.g., the temporal course of potential modulations in cortical excitability. Furthermore, monitoring of TEPs and MEPs during rTMS can reveal how many pulses are necessary to induce an inhibitory or excitatory effect. To the extent of our knowledge, only a handful of studies have focused on online rTMS-EEG analysis.

For example, Hamidi et al. (2010) recorded EEG during 3 s trains of 10 Hz rTMS (30 pulses) administered over the postcentral gyrus and the superior parietal lobule. They could demonstrate an initial decrease followed by an increase in the amplitude of TMS-evoked brain responses with consecutive number of applied pulses. In another study, the left primary motor cortex was stimulated with 40 trains á 10 pulses of 20 Hz rTMS (400 pulses). Based on average electrophysiological responses for 10 pulses (mean over all trains), very early TEP components indicated an increase in amplitude from the first to the tenth pulse (Veniero et al., 2010).

Even though the above-mentioned experiments report EEG changes throughout rTMS, explicit conclusions about the temporal course of cortical dynamics of rTMS protocols cannot be made, since only a few pulses were applied/analyzed. In their study, Helfrich et al. (2012) applied 900 pulses of 1 Hz rTMS over the primary motor cortex in children diagnosed with ADHD and recorded EEG simultaneously. The authors analyzed temporal changes only for the N100 (no other TEP components or MEPs) and observed an amplitude decrease over the course of stimulation which was most pronounced during the first 500 pulses. Thereafter, the N100 amplitude remained relatively stable. This could mean that neuroplastic effects took place at the beginning of the rTMS session and the rest of the pulses could have no additional effect. This study suggests the relevance of adaption of number of pulses of rTMS to clinical settings to avoid over-stimulation or in other cases under-stimulation.

However, it is unclear whether the healthy adult brain exhibits similar alterations and if changes over the course of stimulation are available with other TEP components or MEPs as well. An understanding of the change of electrophysiological metrics (EEG, EMG) during the course of rTMS in healthy subjects is essential prior to its application and interpretation in pathological conditions. Accordingly, the objective of the present study was to shed light on ongoing alterations in cortical reactivity during 1 Hz rTMS applied over the motor cortex in healthy subjects. It is assumed that 1 Hz rTMS decreases cortical excitability (Fitzgerald et al., 2006).

First, due to limited preliminary data we investigated the plausibility and validity of online rTMS-EEG analysis in sensor and source space as well as for online rTMS-EMG analysis. For this purpose, we searched for MEPs and TEPs and also examined cortico-muscular coherence between EEG and EMG metrics. Second, we analyzed changes of rTMS-induced effects during the rTMS course for TEP and MEP amplitudes as well as global neuronal activity. We hypothesized that rTMS effects may vary across individuals and depend on subject-specific physiological parameters as well as their interaction with technical rTMS specifications (Fitzgerald et al., 2006; López-Alonso et al., 2014; Klomjai et al., 2015; Rossini et al., 2015; Guerra et al., 2017; Polanía et al., 2018; Prei et al., 2023). Therefore, we analyzed temporal changes in EEG on single-subject level as well. For this purpose we used the global mean field power (GMFP), a measure of global neuronal activity, since TEPs exhibit high variability between subjects (Ozdemir et al., 2021).

## Materials and Methods

The experimental design and methodological approaches applied in the present study were approved by the local ethics committee (Ethical Approval #21-2662-101). All participants gave written informed consent prior to study participation in accordance with the Declaration of Helsinki.

### Participants

A number of 11 healthy subjects (six female) were recruited in conformity with the following inclusive criteria: German-speaking; right-handedness; age between 18 and 50 years; absence of any severe neurological, psychiatric, or other severe somatic condition; no intake of psychoactive substances or medication as well as no contraindications with respect to TMS such as ferromagnetic implants in the head area; and a severe traumatic brain injury or epileptic seizure in the past. All subjects received a monetary compensation for study participation. In one subject (male, 24 years), EEG data could not be reliably analyzed because of high noise levels. Therefore, this subject was excluded from further analysis. Thus, data from *N* = 10 subjects (six female) aged between 22 and 33 years (*M* = 26.50 ± 3.66 years) was considered for the present analysis.

All subjects were right-handed according to the Edinburgh Handedness Inventory (Oldfield, 1971), showed normal IQ scores in a vocabulary-based test for general intelligence (*M* = 104.20 ± 11.27) (Lehrl, 1999), and reported low to high general physical activity behavior during the last 7 d (low: 1, moderate: 4, high: 5) (Lee et al., 2011). The majority stated to be well rested at the day of the experiment and to have slept from 5 to 8 h the previous night (*M* = 6.27 ± 1.15) (one subject felt not well rested after 7.5 h of sleep). No depressive symptoms were evident in any of the participants using the Major Depression Inventory (*M* = 5.10 ± 2.73; Max = 9.00) (Bech et al., 2001).

### Experimental design

The experiment started with EEG preparations, the determination of subjects’ motor hotspot and resting motor threshold (RMT) and was followed by the administration of 1,000 pulses of 1 Hz rTMS (see section *Transcranial magnetic stimulation*). While rTMS was applied, subjects were requested to sit as still as possible, focus on a fixation cross, avoid head and body movements as well as extensive eye blinks or ocular motions. In parallel to rTMS, EEG and EMG data were recorded continuously (see section *Electrophysiology*). Because TMS pulses are accompanied by loud click noises, which can elicit auditory evoked potentials (Nikouline et al., 1999; ter Braack et al., 2015), a masking noise was delivered through ER-3C 10 Ω Insert Earphones (Etymotic Research Inc.) together with an iPod Touch 7th Generation (Apple Inc.) to minimize auditory coactivation. The masking noise was composed of 70% white noise and 30% TMS click noise and created with the TAAC toolbox (Russo et al., 2022) in MATLAB (MATLAB R2018b; MathWorks). Its loudness level was individually adjusted by the subjects via the application of single TMS pulses prior to rTMS (maximum loudness of 85 dB SPL). After the stimulation, the perception of the TMS click during rTMS was assessed in retrospect by means of a Visual Analogue Scale (VAS) from 1 to 10 (1, not perceptible; 10, very loud). Before and after the experiment, participants further evaluated their level of attention, tiredness and excitement likewise on VASs from 1 to 10 (10, utmost level of the respective scale) and filled out a questionnaire for unintended TMS effects (Giustiniani et al., 2022).

### Transcranial magnetic stimulation

A MagPro X100 stimulator together with MagOption (MagVenture A/S) and an active-cooled figure-of-eight coil (Cool-B65 A/P; MagVenture A/S) was used for magnetic stimulations. Biphasic pulses with an induced current flow in anterior-posterior to posterior-anterior direction were used for all magnetic stimulations. For motor hotspot identification, the electrode C3 (10–20 system; Klem et al., 1999) was used as a starting point with the coil oriented approximately 45° to the sagittal midline with its handle pointing backwards. The subjects’ motor hotspot was defined as the point where single TMS pulses evoked stable MEPs with high amplitude. Once the individual motor hotspot was identified, the coil was fixated in this position with a specific coil holder and subjects’ RMT of the left motor cortex was determined with mounted EEG cap in accordance to the method proposed by Rossini et al. (2015) - the lowest stimulation intensity needed to elicit MEPs of >50 μV peak-to-peak amplitude in 50% of applied TMS pulses. MEPs were derived via EMG recordings of the first dorsal interosseous muscle (FDI) of the right hand (see section *Electrophysiology*). For rTMS, 1000 pulses were administered at a frequency of 1 Hz (110% RMT) over the individual motor hotspot of the left hemisphere.

### Electrophysiology

#### Data acquisition

EEG data were recorded during rTMS using Brain Vision Recorder 1.23 with a BrainAmp DC system (Brain Products GmbH) and a TMS compatible elastic electrode cap (Easycap GmbH) with 64 passive electrodes placed according to the 10–20 system (Klem et al., 1999). The signal was recorded at a sampling rate of 5 kHz and online referenced to FCz (GND: AFz). Impedances were kept below 10 kΩ. EMG activity throughout rTMS as well as for RMT determination was recorded with the same setup together with a BIP2AUX adapter (Brain Products GmbH) from the FDI muscle of the right hand using a bipolar belly-tendon electrode montage (GND: Processus styloideus ulnaris) and adhesive electrodes (15 × 20 mm). Prior to attaching the EMG electrodes, subjects’ skin was prepared using medical alcohol and skin preparation gel.

#### Preprocessing

EEG signals were preprocessed in MATLAB (MATLAB R2018b; MathWorks) using the EEGLAB toolbox (Delorme and Makeig, 2004) together with the TMS-EEG Signal Analyzer extension (TESA; Rogasch et al., 2017). The entire preprocessing procedure followed the suggested TESA example pipeline. Channel C3 was used for TMS artifact detection and labeling. Datasets were segmented into 800 ms epochs, starting from 300 ms before to 500 ms after the TMS pulse. Accordingly, 1,000 given pulses in a rTMS sequence result in 1,000 trials per subject. Posterior channels TP9, TP10, and Iz were excluded from all further preprocessing and analysis steps as these typically provide a low signal-to-noise ratio. Noisy or aberrant electrodes were identified using automatic channel rejection and a *z*-score threshold of *z* = 4 as well as visual inspection and stored for later interpolation. The signal was demeaned via subtracting the average of the entire epoch from each single timepoint. Artifact-contaminated activity around the TMS pulse was liberally removed from −13 to 10 ms and interpolated via cubic interpolation (Thut et al., 2011). In a next step, the data were down-sampled to 1 kHz and trials containing noisy segments such as movements or blinks at the time of TMS pulse were rejected.

A two-step independent components analysis (ICA, fastICA) approach was conducted. Prior each ICA the time window of the TMS artifact was replaced with constant zero amplitudes and was subsequently interpolated using cubic interpolation as well. The first ICA was used to identify and remove components containing TMS-related muscular, movement, or electrical artifacts. In favor of removing further nonneural activity, the EEG signal was bandpass-filtered from 1 to 100 Hz and bandstop-filtered from 48 to 50 Hz (fourth-order Butterworth filters) before a second ICA was conducted to reject all other artifacts not directly related to TMS just as ocular blinks/movements, electrode noise, or persistent muscle activity. Noisy channels were interpolated via spherical spline interpolation (Perrin et al., 1989), and the data were re-referenced using an average reference. The preprocessed EEG data were then exported to fieldtrip (Oostenveld et al., 2011) and underwent a final validation, whereby the data were visually inspected trial-by-trial and remaining artifacts were removed. 10–20% of EEG trials were rejected during preprocessing procedures of the individual subjects, resulting in a total number of 800 artifact-free trials (matched for the whole group) that were used for further analysis with fieldtrip.

The EMG data were preprocessed in MATLAB using the fieldtrip toolbox. The data were resampled to 1 kHz, bandpass-filtered from 10 to 500 Hz using a zero-phase first-order Butterworth filter and detrended.

#### Validity of online rTMS-EEG/EMG analysis

To assess the plausibility of EEG analysis throughout 1 Hz rTMS, a grand average of all 800 artifact-free trials for the EEG channels FC1, FC3, C1, C3, CP3, and CP1 around the stimulated area was calculated. The resulting average waveform was then used to identify the peak activity for the TEP components N15, P30, N45, P60, and N100. The average activity within a ±5 ms time window around the peaks of the N15, P30, N45, and P60 and ±10 ms for the peak of the N100 was then used to inspect the topographical distribution of electrical potentials (µV).

In order to localize cortical generators of sensor activity, source localization via minimum norm estimation (Dale et al., 2000) within the above-mentioned time windows per TEP component was performed. A standard boundary element head model (Oostenveld et al., 2003) and a standard source model (cortex_20484.surf.gii) were deployed. Prior to leadfield calculation, the alignment of the present electrode layout was checked visually for correct positioning over the scalp to avoid placements within the tissue of the head model. Grand averages of source-localized evoked activity within the TEP time windows of interest were then plotted on a 3D brain surface aligned to MNI space (Tzourio-Mazoyer et al., 2002).

Online rTMS-EEG and rTMS-EMG data were classified as valid if typical TEPs (time course, topography, source activity) and typical biphasic MEP waveforms as published in the literature were found at the group level. As part of the validity check, cortico-muscular coherence was analyzed via Spearman correlations of EEG and EMG data. Therefore, the peak-to-peak amplitude (µV) of the biphasic MEP within the time window 20–50 ms post TMS pulse for the 800 artifact-free EEG trials was calculated, averaged over all subjects, and correlated with TEP amplitudes and GMFP.

#### TMS-evoked EEG and EMG quantification for statistical analysis

By means of the above-mentioned topographical electrical potential distributions, an electrode cluster comprised of six sensors (see above) featuring the maximum positive or negative activity (µV) was identified per TEP component (N15, P30, N45, P60, N100). These TEP-specific regions of interest (ROI) were then utilized for TEP amplitude extraction as described in the following.

TEP peaks derived from the average waveform together with a ±5 ms time window for the components N15, P30, N45, and P60, respectively, a ±10 ms time window for the N100 were used as search windows to detect the peak TEP amplitude per subject and trial. In a next step, the average activities (µV) were calculated for the time interval of ±5 ms (N15, P30, N45, P60), respectively, ±10 ms (N100) in relation to the identified TEP peaks per trial and per subject (800 artifact-free trials per subject). As dipole changes in the brain also appear in electrodes distant from the stimulated area, the GMFP can be considered a sensitive quantification of TMS-related excitability of the entire cortex. GMFP was computed for the N15-P30-N45-P60 time period, per trial and subject according to the following formula:

$$\mathrm{GMFP}\;(t)=\;\sqrt{\frac{\left[\sum_{i}^{k}\;{\left(V_{i}(t)-V_{\mathrm{mean}}(t)\right)}^{2}\right]}{K}}\;.$$

Thereby, *t* corresponds to the time of interest, *V* to the voltage in sensor *i*, *K* to the number of sensors, and *V*_mean_ represents the average voltage of all sensors (Esser et al., 2006).

As already mentioned above, for the EMG data the peak-to-peak amplitude (µV) of biphasic MEPs within the time of 20–40 ms after the TMS pulses for all artifact-free EEG trials was computed. TEP and EMG amplitudes and the GMFP for each trial and subject were exported from fieldtrip to R for further statistical analysis.

### Statistical analysis

All statistical analyses were performed with the software R (R version 4.0.3; R Foundation for Statistical Analysis). Statistical significance levels were set at 5% for all analyses.

Pre to post changes in VASs for subjects’ level of attention, tiredness and excitement were assessed with paired samples *t* tests or Wilcoxon tests in case of a violation of statistical assumptions.

### TMS-evoked electrophysiological changes over time

To statistically evaluate potential changes over time respectively changes as a function of the amount of administered rTMS pulses at group level, growth model fitting was performed for each TEP component (using the corresponding ROI) as well as for EMG and GMFP data. First, an (1) intercept model was created with subject as a random effect, which allows intercepts to vary across subjects. Next, (2) the number of Trials (Pulses) was added as a fixed effect to the model, followed by (3) the inclusion of a random slope, which assumes that the effect of Trials (pulses) differs across subjects. As trials which are close in time to each other tend to show a higher correlation than trials distant in time plus the present dataset is dealing with equally spaced datapoints (1 Hz rTMS, 1 s interpulse interval), (4) a first-order autoregressive covariance structure was added to the model. Since potential changes over time do not necessarily have to follow a linear trend, curvilinear trends were also considered in the model fitting procedure. Second- and third-order polynomials were added to the model to examine potential (5) quadratic or (6) cubic trends in the data. By examining different trends in our model fitting approach, we can provide detailed insights into the time pattern of cortical excitability beyond linear time courses e.g., fluctuations over time.

To identify the model with the best fit for the data, the Akaike information criterion (AIC) and Bayesian information criterion (BIC) were checked for a decrease along with a significant model improvement as evaluated via Likelihood Ratio Tests (Harrison et al., 2018).

For analysis of potential differences between e.g., stimulation beginning and ending, as well as to uncover possible plateau effects in TEP/EMG amplitudes or GMFP, linear mixed effect model fitting was applied. For this, 10 blocks of 80 pulses each were formed containing the average activity of 80 trials (B1–B10). Equally to the growth model fitting procedure, Subject was treated as a random effect and Block was added as a predictor. Models were likewise compared with Likelihood Ratio Tests and fixed effects were evaluated via the Expected Mean Square Approach. In case of a significant effect of Block, post hoc Tukey contrasts were performed to examine potential differences between blocks. Post hoc results were adjusted for multiple comparisons using the Tukey method.

The same growth model fitting and linear mixed effect model fitting approaches were used to assess potential changes over time, respectively, changes with the amount of administered rTMS pulses in global cortical excitability on a single-subject level. Single-subject analyses were performed using the GMFP only. A random effect of Subject and the addition of a random slope are omitted in single-subject model fitting proceedings as modeling differences between subjects is not relevant in single-subject analysis.

## Results

RMT (%) was 55.60 ± 3.63 (min = 48, max = 60) on average. Please note that RMT is higher in EEG measurements due to higher cortex-coil distance caused by the wearing of an EEG cap. Mean rTMS stimulation intensity (%) was 61.30 ± 3.95 (min = 53, max = 66). Two subjects reported headache during rTMS persisting after stimulation offset. Two further participants experienced itching on the scalp during rTMS, which ended with stimulation offset. No further rTMS-related side effects were reported. Subjects’ level of attention significantly decreased (pre, *M* = 7.78 ± 0.99; post, *M* = 6.28 ± 1.40; *t*_(9)_ = 6.71, *p* < 0.001), similarly subjects’ level of tiredness increased from pre to post rTMS (pre, *M* = 4.10 ± 1.66; post, *M* = 6.29 ± 2.10; *t*_(9)_ = −2.85, *p* = 0.019). No changes in excitement were observed. The TMS click masking noise had an average loudness of 82.67 ± 2.38 dB SPL (min = 78.15; max = 83.80). The perception of the TMS click during rTMS ranged from 2 to 8 (*M* = 5.10 ± 2.13) on a VAS (1–10).

### Validity evaluation of online rTMS-EEG analysis

**Figure 1.**
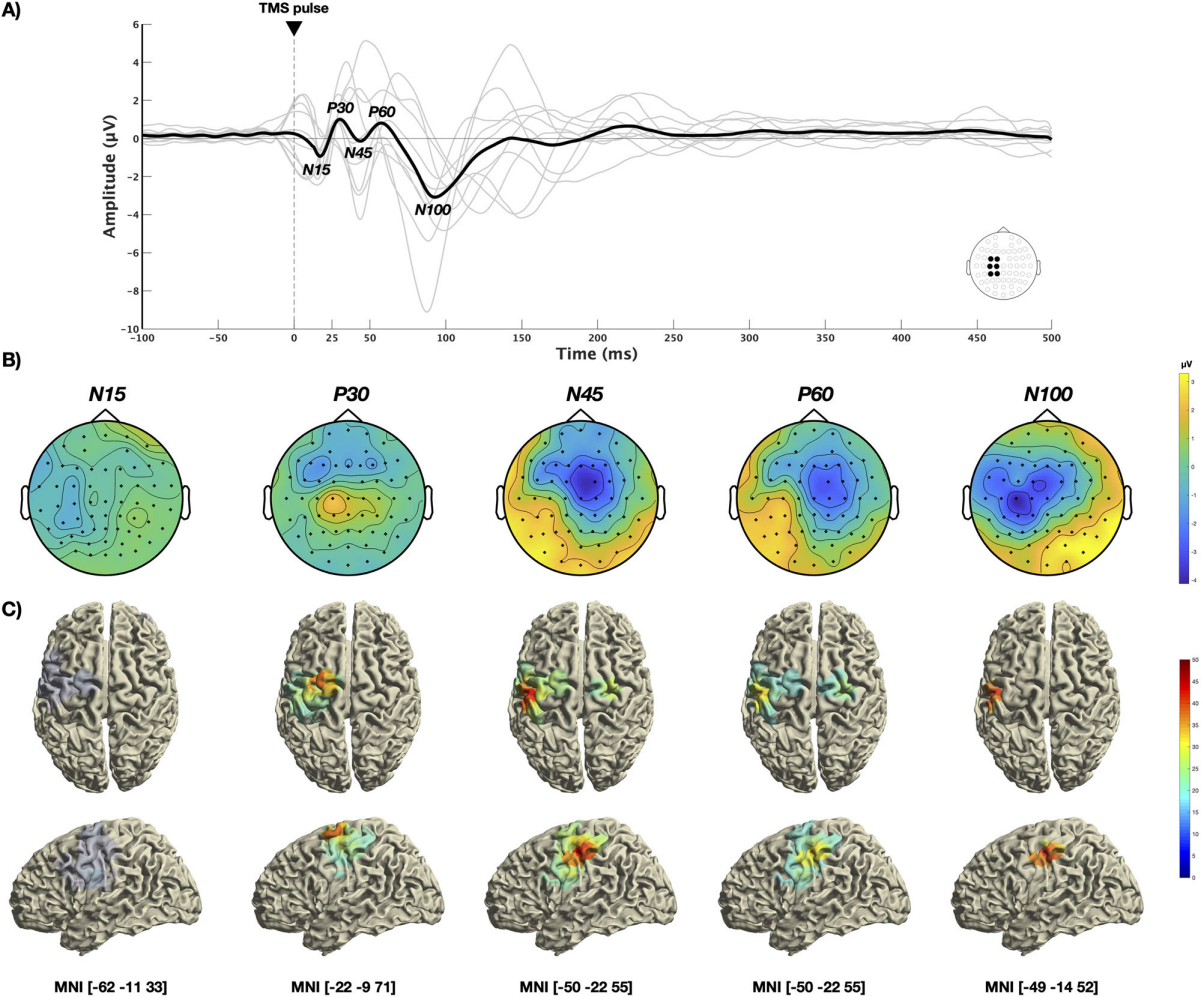
Average sensor and source activity. ***A***, Average waveform of motor cortical activity (FC1, FC3, C1, C3, CP3, CP5) shows typical TEP components – N15 (17 ms), P30 (30 ms), N45 (44 ms), P60 (57 ms), and N100 (93 ms). ***B***, Sensor activity demonstrates distinct topographical distributions of electrical potentials per TEP component with the N15 (12–22 ms) showing maximum average activity over left temporal and parietal areas, the P30 (25–35 ms) over central sensors, the N45 (39–49 ms) over central and right frontal regions, the P60 (52–62 ms) over left parietal electrodes, and the N100 (83–103 ms) over central and left parietal channels. ***C***, Projecting average TEP sensor activity into source space via minimum norm estimation revealed cortical generators in motor and somatosensory cortical regions: N15, left primary motor cortex (BA4); P30, left premotor cortex (BA6); N45, left primary somatosensory cortex (BA1); P60, left primary somatosensory cortex (BA1); N100, left primary somatosensory cortex (BA1).

Figure 1*A* exemplifies the average waveform using a predefined ROI (FC1, FC3, C1, C3, CP1, CP3) for sensor-based motor cortical activity. The amplitudes of the respective TEPs peaked at the following times after the TMS pulse: N15, 17 ms; P30, 30 ms; N45, 44 ms; P60, 57 ms; and N100, 93 ms.

Using these peak times together with a predefined time window as described in the methods section above, the average topographical distribution of electrical potentials was then plotted on sensor level. As can be seen from Figure 1*B* the maximum average activity for the N15 (12–22 ms) appeared over left temporal and parietal areas, the P30 (25–35 ms) peaked over central sensors, the N45 (39–49 ms) peaked over central as well as right frontal areas, whereas the maxima for the P60 (52–62 ms) was present over left parietal electrodes. The maximum amplitude for the N100 (83–103 ms) appeared over central and left parietal channels.

Projecting average TEP sensor activity into source space via MNE exposed cortical generators in motor and somatosensory cortical regions. The maximum source activity for the N15 was localized in the left primary motor cortex (BA4; MNI: −62 −11 33), whereas the P30 was source-localized in the left premotor cortex (BA6; MNI: −22 −9 71). The left primary somatosensory cortex (BA1) was identified as the source for the N45 (MNI: −50 −22 55), the P60 (MNI: −50 −22 55) and the N100 (MNI: −49 −14 52). Source localization results are shown in Figure 1*C* for all TEP components.

Typical biphasic MEPs were observed in EMG recordings of the right hands FDI muscle during rTMS. Cortico-muscular coherence was analyzed via Spearman correlations between EEG and EMG data and is illustrated in Figure 3*B*. Significant but negligible correlations were observed for all TEP components, whereas no significant correlation was present for the GMFP.

### Time course analyses on group level

**Figure 2.**
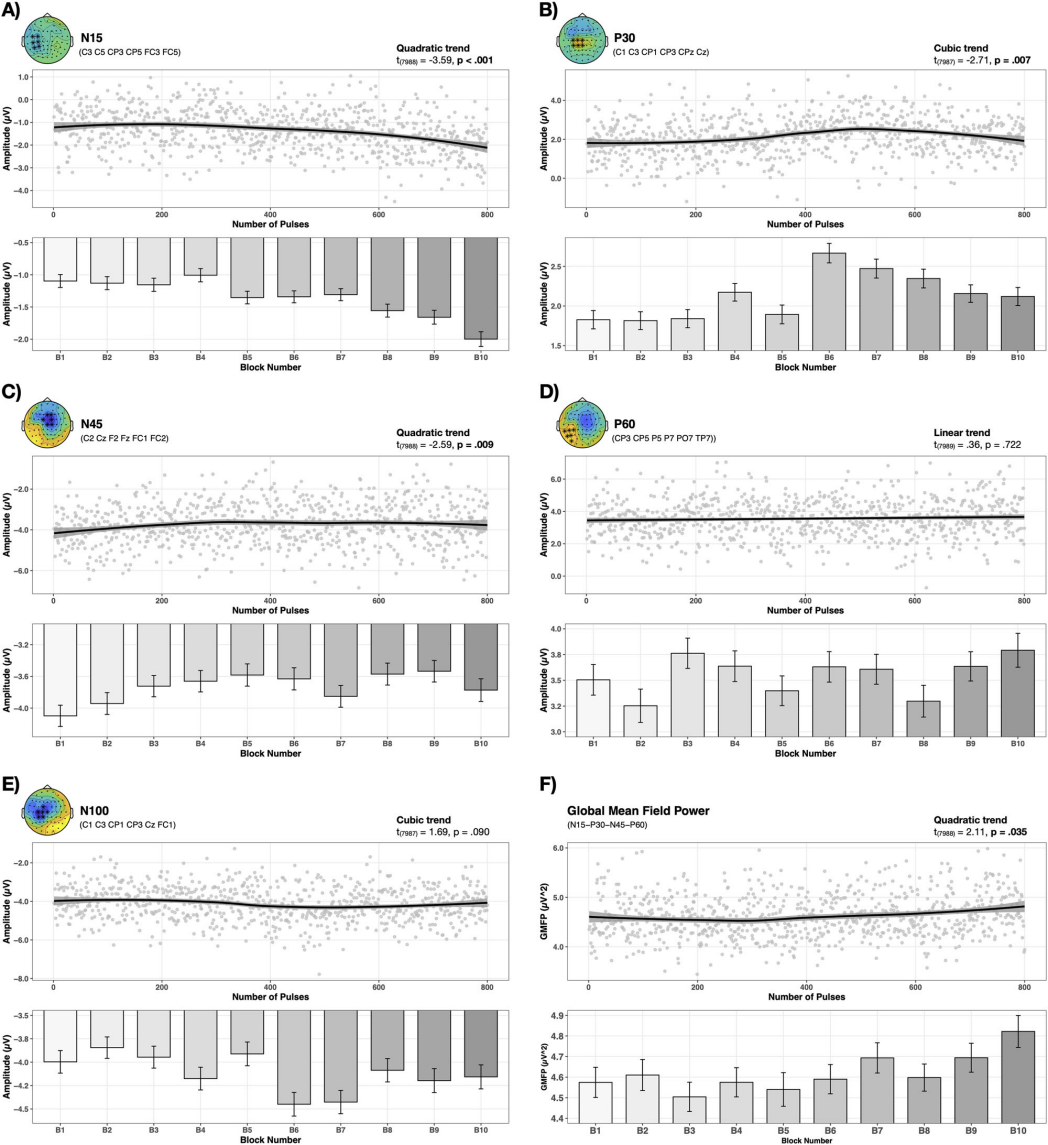
Temporal progression of TEPs and GMFP. Changes during 1 Hz rTMS of the motor cortex are shown for each TEP component and global brain activity (GMFP). Distinct patterns and trends of temporal progression were observed. Gray dots represent single trial data averaged over all subjects. ***A***, N15 amplitude change followed a quadratic trend and showed an increase with ongoing stimulation. ***B***, A cubic trend was present for the P30. Peak amplitude was achieved after approximately 500 pulses which subsequently decreased. ***C***, Temporal change of the N45 was best described by a quadratic trend. N45 amplitude gradually decreased during the first 500 pulses and exhibited a subsequent amplitude increase followed by another decrease and increase on a descriptive level. ***D***, No statistical relevant changes were observed for the P60. ***E***, The N100 temporal progression followed a cubic trend, demonstrated a rapid amplitude increase after about 500 pulses and reached a stable plateau characterized by no statistically significant differences after this rise. ***F***, GMFP followed a quadratic trend and increased with ongoing stimulation.

ROIs featuring maximum activation (µV) are highlighted and listed in Figure 2 per TEP component. These TEP-specific ROIs were then used to evaluate the change in TEP amplitudes over time, respectively, with the amount of given rTMS pulses.

Each growth model with the best fit for the data featured a random slope (differences in fixed effect across subjects) and a first order covariance structure (data points close in time correlate).

In the following, results from growth model and linear mixed effect model fitting are delineated per TEP component as well as for the GMFP and EMG data. Detailed results of post hoc contrasts demonstrating significant differences can be found in Table 1.

**Table 1.** Significant post hoc Tukey contrasts – group level

Contrast	Estimate	*T*_(df, se)_	*p*	Contrast	Estimate	*T*_(df, se)_	*p*
**N15**				**GMFP**			
Block 1–Block 9	0.56	4.02 _(7,981, 0.14)_	0.002	Block 1–Block 10	−0.26	−4.51 _(7,981, 0.06)_	<0.001
Block 1–Block 10	0.90	6.44 _(7,981, 0.14)_	<0.001	Block 2–Block 10	−0.20	−3.41 _(7,981, 0.06)_	0.023
Block 2–Block 9	0.53	3.78 _(7,981, 0.14)_	0.006	Block 3–Block 7	−0.19	−3.31 _(7,981, 0.06)_	0.032
Block 2–Block 10	0.87	6.21 _(7,981, 0.14)_	<0.001	Block 3–Block 9	−0.19	−3.21 _(7,981, 0.06)_	0.044
Block 3–Block 9	0.50	3.60 _(7,981, 0.14)_	0.012	Block 3–Block 10	−0.28	−4.91 _(7,981, 0.06)_	<0.001
Block 3–Block 10	0.84	6.02 _(7,981, 0.14)_	<0.001	Block 4–Block 10	−0.19	−3.35 _(7,981, 0.06)_	0.028
Block 4–Block 8	0.55	3.92 _(7,981, 0.14)_	0.004	Block 5–Block 10	−0.21	−3.68 _(7,981, 0.06)_	0.028
Block 4–Block 9	0.65	4.66 _(7,981, 0.14)_	<0.001	Block 8–Block 10	−0.19	−3.25 _(7,981, 0.06)_	0.038
Block 4–Block 10	0.99	7.08 _(7,981, 0.14)_	<0.001	**EMG**			
Block 5–Block 10	0.65	4.61 _(7,981, 0.14)_	<0.001	Block 1–Block 4	−92.17	−6.66 _(7,981, 13.8)_	<0.001
Block 6–Block 10	0.66	4.69 _(7,981, 0.14)_	<0.001	Block 1–Block 5	−68.62	−4.96 _(7,981, 13.8)_	<0.001
Block 7–Block 10	0.69	4.93 _(7,981, 0.14)_	<0.001	Block 1–Block 6	−76.67	−5.54 _(7,981, 13.8)_	<0.001
**P30**				Block 1–Block 7	−105.68	−7.64 _(7,981, 13.8)_	<0.001
Block 1–Block 6	−0.84	−5.61 _(7,981, 0.15)_	<0.001	Block 1–Block 8	−84.33	−6.10 _(7,981, 13.8)_	<0.001
Block 1–Block 7	−0.65	−4.31 _(7,981, 0.15)_	<0.001	Block 1–Block 10	−53.97	−6.10 _(7,981, 13.8)_	0.004
Block 1–Block 8	−0.52	−3.47 _(7,981, 0.15)_	0.019	Block 2–Block 4	−109.83	−7.94 _(7,981, 13.8)_	<0.001
Block 2–Block 6	−0.85	−5.69 _(7,981, 0.15)_	<0.001	Block 2–Block 5	−86.29	−6.24 _(7,981, 13.8)_	<0.001
Block 2–Block 7	−0.66	−4.39 _(7,981, 0.15)_	<0.001	Block 2–Block 6	−94.34	−6.82 _(7,981, 13.8)_	<0.001
Block 2–Block 8	−0.53	−3.55 _(7,981, 0.15)_	0.014	Block 2–Block 7	−123.35	−8.92 _(7,981, 13.8)_	<0.001
Block 3–Block 6	−0.83	−5.52 _(7,981, 0.15)_	<0.001	Block 2–Block 8	−102.00	−7.37 _(7,981, 13.8)_	<0.001
Block 3–Block 7	−0.63	−4.22 _(7,981, 0.15)_	<0.001	Block 2–Block 9	−57.59	−4.16 _(7,981, 13.8)_	0.001
Block 3–Block 8	−0.51	−3.28 _(7,981, 0.15)_	0.025	Block 2–Block 10	−71.64	−5.18 _(7,981, 13.8)_	<0.001
Block 4–Block 6	−0.49	−3.30 _(7,981, 0.15)_	<0.033	Block 3–Block 4	−61.71	−4.46 _(7,981, 13.8)_	<0.001
Block 5–Block 6	−0.77	−5.16 _(7,981, 0.15)_	<0.001	Block 3–Block 6	−46.21	−3.34 _(7,981, 13.8)_	0.029
Block 5–Block 7	−0.58	−3.86 _(7,981, 0.15)_	0.005	Block 3–Block 7	−75.22	−5.44 _(7,981, 13.8)_	<0.001
Block 6–Block 9	−0.51	−3.41 _(7,981, 0.15)_	0.023	Block 3–Block 8	−53.87	−3.89 _(7,981, 13.8)_	0.004
Block 6–Block 10	−0.55	−3.65 _(7,981, 0.15)_	0.001	Block 4–Block 9	52.25	3.78 _(7,981, 13.8)_	0.006
**N45**				Block 7––Block 9	65.76	4.75 _(7,981, 13.8)_	<0.001
Block 1–Block 8	−0.53	−3.14 _(7,981, 0.17)_	0.050	Block 7–Block 10	51.71	3.74 _(7,981, 13.8)_	0.007
Block 1–Block 9	−0.84	−3.36 _(7,981, 0.17)_	0.027	Block 8–Block 9	44.41	3.21 _(7,981, 13.8)_	0.044
**N100**							
Block 2–Block 6	0.60	4.18 _(7,981, 0.15)_	0.001				
Block 2–Block 7	0.58	4.02 _(7,981, 0.15)_	0.002				
Block 3–Block 6	0.51	3.48 _(7,981, 0.15)_	0.018				
Block 3–Block 7	0.48	3.32 _(7,981, 0.15)_	0.031				
Block 5–Block 6	0.54	3.72 _(7,981, 0.15)_	0.008				
Block 5–Block 7	0.52	3.56 _(7,981, 0.15)_	0.014				

GMFP, global mean field power; EMG, electromyography; df, degrees of freedom; se, standard error.

Growth model fitting for the N15 revealed that a quadratic trend best described the pattern in the data over time with a significant effect of Trials (*t*_(7,988)_ = −3.59, *p* < 0.001), indicating that the N15 amplitude changes with the number of given rTMS pulses (cf. Fig. 2*A*). Likewise, a significant effect of Block was present with ensuing post hoc contrasts showing significant differences between the blocks B1, B2, B3, B4 versus B9, B10. Further differences between B4 versus B8 and B5, B6, B7 versus B10 were observed. Thereby first-mentioned blocks always demonstrated lower amplitudes, indicating a negative amplitude increase over time for the N15 (cf. Fig. 2*A* and Table 1).

As can be seen from Figure 2*B*, a cubic trend had the best fit for the P30 data. The P30 amplitude significantly changed over time (*t*_(7,987)_ = −2.71, *p* = 0.007) as per a second-order polynomial. Post hoc tests revealed significant differences between the blocks B1, B2, B3 versus B6, B7, B8 as well as B4 versus B6 and B5 versus B6, B7 with the first-mentioned always showing lower amplitude. Moreover, significant differences for B6 versus B9, B10 were observed, whereby the amplitude of B6 appeared to be higher. Accordingly, the peak amplitude of the P30 was reached at B6 and subsequently declined (cf. Fig. 2*B* and Table 1).

The pattern in the data for the N45 was best described by a quadratic trend and featured a significant effect of Trials (*t*_(7,988)_ = −2.59, *p* = 0.009) (cf. Fig. 2*C*). During the first half of the rTMS intervention the amplitude of the N45 gradually decreased, before it increased again and subsequently decreased once more. Significant differences between B1 versus B8, B9 were present for the N45, whereas B1 indicated a higher negative amplitude than B8 and B9 (cf. Fig. 2*C* and Table 1).

For the P60, the model featuring a linear trend had the best fit for the data. However, no significant effect of Trial was present. No effect of Block was observed for the P60 in our linear mixed effect fitting approach (cf. Fig. 2*D*).

The model accounting for a cubic trend had the best fit for the N100 amplitude changes with given number of pulses. A statistical trend for an effect of Trial was observed (*t*_(7,987)_ = 1.69, *p* = 0.090) (cf. Fig. 2*E*). Post hoc contrast for the N100 exposed significant differences for B2, B3, B5 versus B6, B7, whereby B6 and B7 featured a higher amplitude indicating an increase in N100 amplitude (cf. Fig. 2*E* and Table 1).

Progression over time in global cortical excitability quantified by the GMFP followed a quadratic trend as specified by our model fitting procedure (cf. Fig. 2*F*). The GMFP significantly changed over time (*t*_(7,988)_ = 2.11, *p* = 0.035) and significant differences were observed for B1, B2, B3, B4, B5, and B8 versus B10 as well as for B3 versus B7, B9, and B10 (cf. Fig. 2*F* and Table 1). Despite initial fluctuations, global neuronal activity increased with the number of applied pulses.

**Figure 3.**
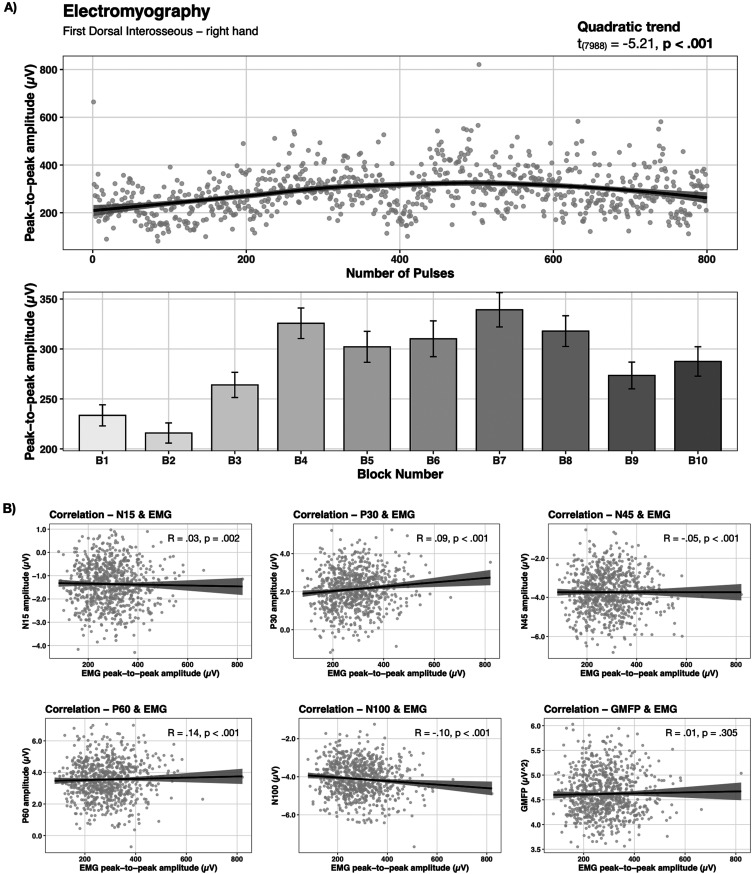
Temporal progression of EMG and cortico-muscular coherence. MEPs were derived from the FDI muscle of the right hand during 1 Hz rTMS of the motor cortex. Gray dots represent single trial data averaged over all subjects. ***A***, The temporal course of the peak-to-peak amplitude followed a quadratic trend and showed its maximum amplitude after approximately 600 given pulses. ***B***, Spearman correlations of MEP peak-to-peak amplitude with TEP components showed significant but negligible correlations. No significant correlation was present for the GMFP.

Alteration of the EMG amplitude was best described by the model accounting for a quadratic trend in the data (cf. Fig. 3*A*) and featured a significant effect of trial (*t*_(7,988)_ = −5.21, *p* < 0.001). EMG amplitudes were significantly lower for B1, B2, and B3 versus B4, B6, B7, and B8 as indicated by post hoc contrasts. Further, significant lower amplitudes for B1 and B2 versus B5 and B10 and B2 versus B9 were present. Moreover, significant differences were observed for B4 versus B9 and B7 versus B9 and B10 as well a B8 versus B9, whereby the amplitude of former blocks appeared to be higher. Accordingly, EMG amplitude increased over the course of given pulses and peaked at B7 followed by a decline (cf. Fig. 3*A* and Table 1).

### Single-subject-level analysis

**Figure 4.**
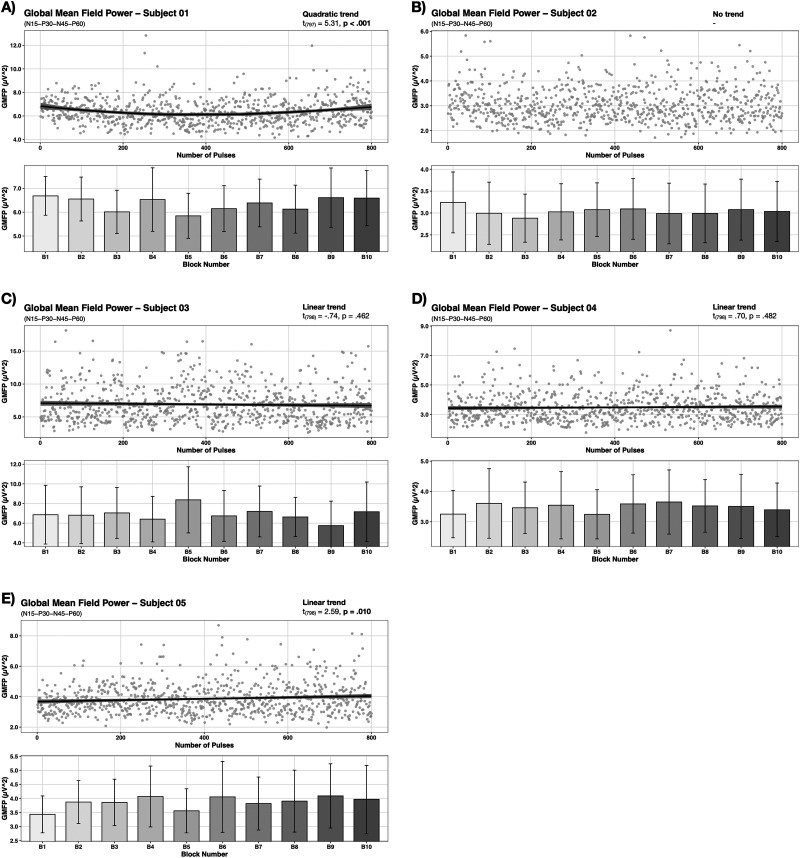
Individual subject temporal progression of GMFP (Subjects 01–05). Changes in GMFP during 1 Hz rTMS of the motor cortex are illustrated for the subjects 01–05. Gray dots represent single trial data per subject. Individual trends of temporal progressions and subject-specific peaks and valleys of GMFP were observed.

**Figure 5.**
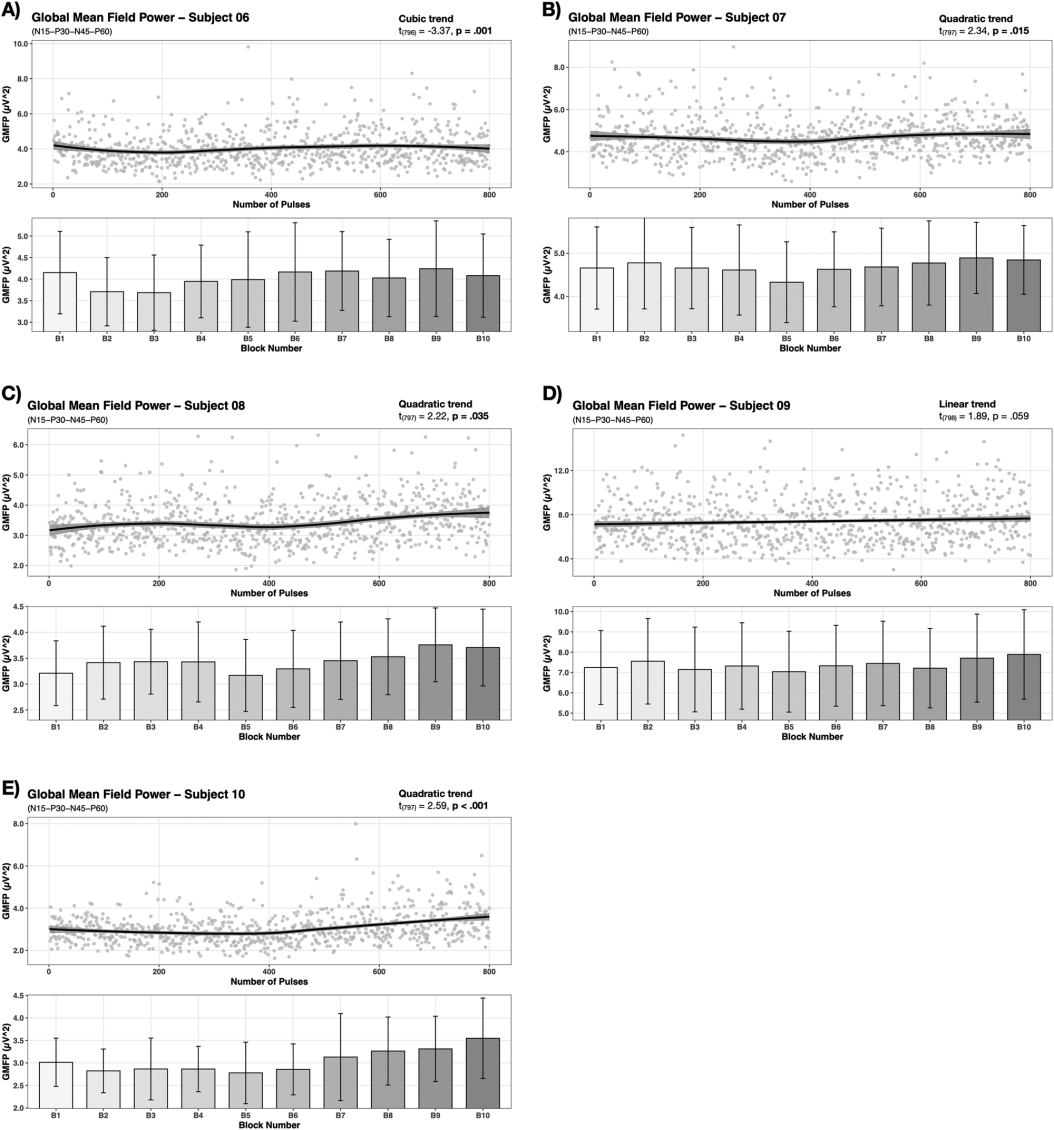
Individual subject temporal progression of GMFP (Subject 06–10). Changes in GMFP during 1 Hz rTMS of the motor cortex are illustrated for the subjects 06–10. Individual trends of temporal progressions and subject-specific peaks and valleys of GMFP were observed.

Single-subject changes in global cortical excitability over the course of rTMS were assessed via GMFP. Equally to our group-level analysis, each growth model with the best fit for the data included a first order covariance structure. Figures and significant post hoc findings for single-subject-level analysis can be found in Figures 4 and 5 and Table 2, respectively. For 4 subjects, a linear trend was identified to have the best fit for the data (cf. Figs. 4*C,D,E*, 5*D*), whereas only in one of which a significant effect of trial was observed (S05: *t*_(798)_ = 2.59, *p* = 0.010) (cf. Fig. 4*E*). In another 4 subjects the model accounting for a quadratic trend best described the trend in the data (cf. Figs. 4*A*, 5*B,C,E*). For all of them a significant effect of trial was evident (S01: *t*_(797)_ = 5.31, *p* < 0.001; S07: *t*_(797)_ = 2.34, *p* = 0.015; S08: *t*_(797)_ = 2.22, *p* = 0.035; S10: *t*_(797)_ = 2.59, *p* < 0.001). A cubic trend in the data as well as a significant effect of trial was identified for one subject (S06: *t*_(797)_ = −3.37, *p* = 0.001) (cf. Fig. 5*A*). Significant effects of block were observed for the subjects S01, S03, S05, S06, S07, S08, S10 by linear mixed effect model fitting. Significant post hoc contrasts can be found in Table 2.

**Table 2.** Significant post hoc Tukey contrasts – single-subject level

Contrast	Estimate	*T*_(df, se)_	*p*	Contrast	Estimate	*T*_(df, se)_	*p*
**GMFP - Subject 01**				**GMFP - Subject 07**			
Block 1–Block 3	0.67	4.07 _(790, 0.16)_	0.002	Block 5–Block 9	−0.56	−3.81 _(790, 0.16)_	0.006
Block 1–Block 5	0.83	5.09 _(790, 0.16)_	<0.001	Block 5–Block 10	−0.52	−3.50 _(790, 0.16)_	0.018
Block 1–Block 6	0.53	3.25 _(790, 0.16)_	0.040	**GMFP - Subject 08**			
Block 1–Block 8	0.56	3.38 _(790, 0.16)_	0.026	Block 1–Block 9	−0.55	−4.87 _(790, 0.16)_	<0.001
Block 2–Block 3	0.54	3.27 _(790, 0.16)_	0.037	Block 1–Block 10	−0.50	−4.41 _(790, 0.16)_	<0.001
Block 2–Block 5	0.71	4.29 _(790, 0.16)_	<0.001	Block 5–Block 8	−0.36	−3.20 _(790, 0.16)_	0.046
Block 3–Block 9	−0.60	−3.63 _(790, 0.16)_	0.004	Block 5–Block 9	−0.59	−5.25 _(790, 0.16)_	<0.001
Block 3–Block 10	−0.58	−3.50 _(790, 0.16)_	0.011	Block 5–Block 10	−54	−4.79 _(790, 0.16)_	0.001
Block 4–Block 5	0.68	4.15 _(790, 0.16)_	0.002	Block 6–Block 9	−0.46	−4.12 _(790, 0.16)_	0.002
Block 5–Block 7	−0.54	−3.29 _(790, 0.16)_	0.035	Block 6–Block 10	−0.41	−3.66 _(790, 0.16)_	0.010
Block 5–Block 9	−0.76	−4.66 _(790, 0.16)_	<0.001	**GMFP - Subject 10**			
Block 5–Block 10	−0.74	−4.52 _(790, 0.16)_	<0.001	Block 1–Block 10	−0.53	−4.84 _(790, 0.16)_	<0.001
**GMFP - Subject 03**				Block 2–Block 8	−0.44	−4.00 _(790, 0.16)_	0.003
Block 1–Block 5	−1.51	−3.53 _(790, 0.16)_	0.016	Block 2–Block 9	−0.49	−4.43 _(790, 0.16)_	<0.001
Block 2–Block 5	−1.56	−3.66 _(790, 0.16)_	0.010	Block 2–Block 10	−0.72	−6.57 _(790, 0.16)_	<0.001
Block 4–Block 5	−1.97	−4.59 _(790, 0.16)_	<0.001	Block 3–Block 8	−0.40	−3.62 _(790, 0.16)_	0.012
Block 5–Block 6	1.63	3.81 _(790, 0.16)_	0.006	Block 3–Block 9	−0.45	−4.05 _(790, 0.16)_	0.002
Block 5–Block 8	1.74	4.07 _(790, 0.16)_	0.002	Block 3–Block 10	−0.68	−6.19 _(790, 0.16)_	<0.001
Block 5–Block 9	2.62	6.12 _(790, 0.16)_	<0.001	Block 4–Block 8	−0.40	−3.63 _(790, 0.16)_	0.011
Block 7–Block 9	1.44	3.37 _(790, 0.16)_	0.027	Block 4–Block 9	−0.45	−4.06 _(790, 0.16)_	0.002
Block 9–Block 10	−1.41	−3.30 _(790, 0.16)_	0.034	Block 4–Block 10	−0.68	−6.20 _(790, 0.16)_	<0.001
**GMFP - Subject 05**				Block 5–Block 7	−0.35	−3.19 _(790, 0.16)_	0.047
Block 1–Block 4	−0.63	−4.04 _(790, 0.16)_	0.002	Block 5–Block 8	−0.49	−4.41 _(790, 0.16)_	<0.001
Block 1–Block 6	−0.62	−3.95 _(790, 0.16)_	0.003	Block 5–Block 9	−0.53	−4.84 _(790, 0.16)_	0.001
Block1–Block 9	−0.66	−4.17 _(790, 0.16)_	0.001	Block 5–Block 10	−0.77	−6.98 _(790, 0.16)_	<0.001
Block 1–Block 10	−0.54	−3.40 _(790, 0.16)_	0.025	Block 6–Block 9	−0.46	−4.13 _(790, 0.16)_	0.002
Block 4–Block 5	0.51	3.23 _(790, 0.16)_	0.042	Block 6–Block 10	−0.69	−6.27 _(790, 0.16)_	<0.001
Block 5–Block 9	−0.53	−3.36 _(790, 0.16)_	0.028	Block 7–Block 10	−0.42	−3.88 _(790, 0.16)_	0.006
**GFMP - Subject 06**							
Block 2–Block 9	−0.53	−3.49 _(790, 0.16)_	0.018				
Block 3–Block 7	−0.50	−3.29 _(790, 0.16)_	0.035				
Block 3–Block 9	−0.55	−3.64 _(790, 0.16)_	0.011				

GMFP, global mean field power; df, degrees of freedom; se, standard error.

## Discussion

The main objective of the present study was to monitor and analyze cortical dynamics via electrophysiological metrics throughout 1 Hz rTMS over the healthy motor cortex. First, we aimed for a plausibility check of such online rTMS-EEG and EMG examinations in sensor and source space. Second, we analyzed the time course of electrophysiological measures on group level (TEPs, MEPs, GMFP) and single-subject level (GMFP).

The average TEP waveform of 1 Hz rTMS corresponds to TEPs of single pulse TMS-EEG datasets which typically use a lesser amount of pulses with an interstimulus interval far below 1 Hz (about 0.1 Hz) (Ilmoniemi and Kicić, 2010; Farzan et al., 2016; Julkunen et al., 2022). The topographical distribution of electrical potentials follows a pattern in accordance with previous studies as well, featuring peak activity of the N15 in the stimulated area, followed by a movement of the maximum amplitude for the P30 into central regions and to more contralateral sensors for the N45. The P60 and the N100 indicated a topographical distribution of the peak amplitude in parietal and centro-parietal sensors of the left hemisphere (Tremblay et al., 2019; Belardinelli et al., 2021; Zazio et al., 2021). This inter-region, respectively, transcallosal activity spread from the stimulated motor cortex to other brain areas highlights the fact that the motor cortex does not operate in isolation but rather it is functionally connected to other brain regions as well (Kricheldorff et al., 2022; Guidali et al., 2023).

Source localization using MNE exposed cortical generators in the primary motor cortex for the N15, in the premotor cortex for the P30 and in the primary somatosensory cortex for the N45, P60, and N100 similar to TEP source estimations already described in the literature (Petrichella et al., 2017; Ahn and Fröhlich, 2021).

It is assumed that late TEP components (50–180 ms) represent auditory coactivation due to the loud click noise accompanied by TMS pulses (Nikouline et al., 1999; ter Braack et al., 2015; Ross et al., 2022) as for example shown by source localizations of late TEP components (100–280 ms) in the auditory cortex (Ahn and Fröhlich, 2021). We only identified non-auditory cortical generators for late TEPs, hence auditory coactivation can be neglected. We observed typical biphasic MEP waveforms throughout rTMS in our EMG recordings, though EMG activity and EEG activity (TEPs, GMFP) were not found to be associated.

In view of the accordance with past observations as well as the plausibility of the present findings in sensor and source space, EEG as well as EMG analyses during 1 Hz rTMS with the available data can be considered as feasible. Just as previous investigations (Helfrich et al., 2012; Biabani et al., 2019; Roos et al., 2021; Zhou et al., 2022), we did not observe any meaningful correlation between cortical and peripheral measures even though we recorded as substantial larger number of trials than previous experiments (cf. Hernandez-Pavon et al., 2023).

Monitoring cortical dynamics during 1 Hz rTMS unveiled different temporal alterations depending on the TEP component of interest. As there is only one comparable study evaluating the changes in cortical excitability during rTMS, a discussion and classification of the present findings is rather difficult. In contrast to Helfrich et al. (2012) who demonstrated a N100 decrease, we observed a rapid N100 increase after approximately 500 pulses (8 min of stimulation), followed by a stable plateau characterized by no statistical differences after this expeditious rise.

Since the N100 has been proposed to signify motor cortical inhibition as well as to indicate modulation in GABAergic inhibition (Casula et al., 2014; Premoli et al., 2014; Roos et al., 2021; Zhou et al., 2022), our present finding suggests that after approximately 8 min of 1 Hz rTMS the level of strongest inhibition is already reached.

The discrepancy in the time course of the N100 compared to the study by Helfrich et al. (2012) may be due to incongruencies in investigated samples (children with ADHD vs healthy adults) as it is suspected that rTMS effects are state-dependent (Silvanto and Pascual-Leone, 2008) and in ADHD pathological patterns of baseline EEG activity were found (Lenartowicz and Loo, 2014; Vahid et al., 2019) and ADHD is associated with dopaminergic dysfunction (Tripp and Wickens, 2009). At this point it needs to be mentioned that the careful elimination of artifacts led to a 20% uncertainty of the exact time course in our data, since 800 artifact-free out of 1,000 available trials were analyzed.

However, insights from our and Helfrich's study are in favor of considerations of dose-effect dependencies in rTMS research (Gamboa et al., 2010; Nettekoven et al., 2014; Schoisswohl et al., 2019; McCalley et al., 2021), as it seems that neuroplastic effects already occurred after the application of approximately 500 pulses respectively 8 min of rTMS and that the remaining pulses induced no additional neuroplastic effect. The proposed underlying mechanisms of neuroplasticity are LTP or LTD of synaptic strength (Chervyakov et al., 2015; Lefaucheur et al., 2020; Jannati et al., 2023). Short phases of LTP or LTD were already found after single rTMS sessions of e.g., 600 pulses (Klomjai et al., 2015) akin to our observation. Evidence in favor of dose-response dependencies also comes from concurrent TMS and functional magnetic resonance imaging demonstrating activity changes with respect to stimulation duration or intensity (cf. Siebner et al., 2022).

A similar progression over time as for the N100 was existent for the P30, with its peak activation appearing after about 8 min of stimulation (500 pulses) as well. The progression of the N45 amplitude is almost opposite to the P30. The N45 gradually decreased during the first half of stimulation and reached its (second) lowest negative amplitude after approximately 8 min of rTMS application. Both components were associated with peripheral measures of motor activation (Mäki and Ilmoniemi, 2010; Gedankien et al., 2017), as demonstrated for instance by Ahn and Fröhlich (2021) via a positive correlation for the P30 and a negative correlation for the N45 with MEPs. Thus, the highest peripheral muscle activation should occur when the P30 amplitude is in its highest positive state and the N45 amplitude in its lowest state. Indeed, in our study the temporal course of P30/N45 is in correspondence with MEP amplitude changes over time.

For the N15, we observed a quadratic change over time, whereby the amplitude increased with ongoing stimulation and reached its maximum at the end of stimulation. The N15 is discussed as a parameter of direct cortical activation of the targeted area (Ilmoniemi and Kicić, 2010; Tremblay et al., 2019), hence our observation might indicate that the activation of the targeted motor cortical area is highest at the end of stimulation. Interestingly and in contrast to the commonly accepted heuristic that low frequency rTMS (≤1 Hz) evokes inhibition and high frequency rTMS (≥5 Hz) causes excitation (Maeda et al., 2000; Fitzgerald et al., 2006) as typically measured via MEPs, we observed an facilitating effect of 1 Hz rTMS. MEP amplitudes increased throughout stimulation and reached its maxima after about 10 min of stimulation (600 pulses). Despite a slight up and down, MEP amplitudes were significantly higher at the end of stimulation in contrast to stimulation start. This finding questions the validity of low frequency rTMS being inhibitory just like other previous investigations (Berardelli et al., 1999; Modugno et al., 2003; Brighina et al., 2005; Daskalakis et al., 2006; Prei et al., 2023).

Moreover, we did not observe any statistical relevant changes in the P60 amplitude. Together with the N45, the P60 is assumed to reflect somatosensory feedback or rather afferent feedback from the hand muscle (Rogasch et al., 2013; Ahn and Fröhlich, 2021). Interpretation as somatosensory feedback would be consistent with our localization of cortical generators for the N45/P60 in the left primary somatosensory cortex but is limited by the absence of any statistical relationship of EEG and EMG measures.

From the present body of literature, it remains unclear which TEP component is most appropriate to track rTMS-related modulations in cortical inhibition or excitation (Casula et al., 2014; Goldsworthy et al., 2021; Voineskos et al., 2021; Zhou et al., 2022). For example the N15 is suspected to reflect direct cortical excitation of the stimulated area (Ilmoniemi and Kicić, 2010; Tremblay et al., 2019), the P30 and the N45 were associated with peripheral measures of cortical excitability (Mäki and Ilmoniemi, 2010; Gedankien et al., 2017) or the N100 is debated to represent cortical inhibition as 1 Hz rTMS increased its amplitude (Casula et al., 2014; Zhou et al., 2022). In the case of the N100, no correlation with MEPs is available in the literature (Casula et al., 2014; Zhou et al., 2022), which potentially hampers its interpretability as a marker for inhibitory or excitatory effects of rTMS, besides commonly accepted MEP amplitudes. On the other hand, it has been discussed that TEPs provide a more sensitive direct readout of TMS effects on the brain than EMG, in spite of missing behavioral correlations (Thut and Pascual-Leone, 2010; Roos et al., 2021). How specific TEP components can be interpreted with respect to inhibitory or excitatory rTMS effects as well as if associations with MEPs and their magnitude are a suitable method to validate TEPs as in some cases TEPs also emerge in the absence of MEPs (Casula et al., 2014; Zhou et al., 2022) needs to be clarified in future studies.

Taken together, we observed temporal progressions and fluctuations of cortical activity depending on the TEP component of interest. Therefore, an index of global cortical activation such as the GMFP might provide more suitable insights into the temporal dynamics appearing during rTMS. Our analysis demonstrated that the temporal progression of GMFP followed a quadratic trend and increased with ongoing stimulation, thus global brain activity increased over the course of 1 Hz rTMS. For the N15 and MEPs an almost identical progression as for the GMFP was observed – overall increase throughout rTMS as per a second-order polynomial. In a study by Fecchio et al. (2017), it was shown that larger MEP amplitudes were associated with larger overall TEP amplitude, similar to our findings of an identical temporal course of MEPs and GMFP.

Past research stresses that rTMS effects are subject to a complex interplay of several technical and subject-specific parameters (López-Alonso et al., 2014; Guerra et al., 2017; Polanía et al., 2018) with several investigations not reporting inhibitory consequences in case of low frequency rTMS (Berardelli et al., 1999; Modugno et al., 2003; Brighina et al., 2005; Daskalakis et al., 2006; Prei et al., 2023). On that account we aimed for an individual subject analysis of GMFP changes. Our findings indicate inter-individual trajectories throughout 1 Hz rTMS with subject-specific peaks and valleys of global neuronal activity with some subjects also demonstrating no changes. The present observations are in accordance with past research reporting inter-individual variability of rTMS responses (Klomjai et al., 2015; Terranova et al., 2019; Prei et al., 2023) and endorse attempts in customizing rTMS protocols for e.g., personalized brain stimulation treatments.

Monitoring and analyzing online rTMS effects with metrics of cortical activation in single sessions prior treatment could serve as a potential novel attempt to adjust brain stimulation protocols according to the individual doses necessary for cortical activation – optimal number of pulses per individuum.

Suspected subject-specific sources of interindividual variability are for example intrinsic brain states, genetic polymorphisms or individual cortical geometries – these as well as the interaction of technical and subject-specific factors could potentially be accountable for our observed inter-individual trends and time courses in evoked global brain activity (Polanía et al., 2018).

Efforts in rTMS personalization for therapeutic use are already pursued and have the potential to account for certain factors of variability such as the individual intrinsic brain state (Cocchi and Zalesky, 2018; Schoisswohl et al., 2020, 2021, 2022; Chou et al., 2021; Klooster et al., 2021).

Because we observed different temporal patterns of electrophysiological measures as well as trends for a decline or increase after peak activation, it would be worthwhile to further investigate how metrics of cortical excitability evolve with the application of a higher quantity of pulses as typically deployed in therapeutic rTMS paradigms.

The distinct topographical peak activity for different TEP components in the present data emphasizes the consideration of individual sensors per TEP component in prospective analysis rather than a single cluster of electrodes for all TEP components like in many past studies.

The present study is also subject to some limitations worth mentioning. As the main focus of the study at hand lies on the feasibility of online rTMS-EEG analysis, as well as on analyzing the time course of electrophysiological data on a whole group and singlesubject level, a rather small sample size was investigated limiting interpretability and generalizability. Hence, the present findings, in particular correlation results of EEG and EMG metrics, should be interpreted with caution. The solely focus on FDI muscles in the present work further limits the interpretability of EMG findings, since MEPs are likely to engage other muscles beyond the FDI as well.

Subjects’ increase in tiredness and decrease in attention could also have influenced the present findings. For example it was demonstrated that during the transition from wakefulness to sleep the quality of TEPs changes e.g., amplitude increase in early TEP components (Massimini et al., 2005). However, any neuromodulation experiment, usually performing protracted stimulation paradigms, might be affected by drowsiness up to a certain extent.

It should be mentioned that insights about time courses in TMS-induced electrophysiological activity of the present study do not come from real-time monitoring, rather after preprocessing and analyses, and should therefore be treated as post hoc sorted online effects. Real-time monitoring of TMS-EEG refers to immediate read-outs of neural activity during the experiment and requires sophisticated technical approaches and dedicated software solutions (Zrenner et al., 2018; Hassan et al., 2022; Hernandez-Pavon et al., 2023).

The intention of the present study setting was to emulate procedures which are typically used in a regular clinical rTMS treatment application. Even if subjects were instructed to avoid head and body movements, potential shifts of the coil position while stimulation cannot be ruled out and might have biased our findings since TMS effects tend to be highly spatially specific. The use of neuronavigation would facilitate a follow-up of potential changes in coil placement. But only a combination with collaborative robots allows for high precision and stability in coil placement together with an online adjustment for subjects’ head movements (Goetz et al., 2019). A replication of the present study under laboratory conditions using a neuronavigated collaborative robot system would be of particular interest.

## Conclusion

The primary objectives of the present experiment were to assess the validity of online rTMS-EEG and rTMS-EMG analyses as well as to monitor the temporal course of TMS-evoked electrophysiological activity throughout 1 Hz rTMS over the healthy motor cortex. We observed typical and plausible TEP sensor and source activity as well as typical biphasic MEP waveforms in accordance with past single pulse TMS investigations. Furthermore, we demonstrate heterogeneous temporal progressions and fluctuations as well as effect saturations depending on the TEP component of interest. Overall, global brain activity increased with the quantity of pulses applied. Inter-individual differences in temporal courses of global neuronal activity were observed. Taken together, our findings are in favor of dose-response considerations as well as attempts in personalization of rTMS protocols.

## Ethics Statement

The study was conducted according to the guidelines of the Declaration of Helsinki and approved by the Ethics Committee of the University of Regensburg, Germany (Ethical Approval #21-2662_1-101). Informed consent was obtained from all subjects involved in the study.

## Synthesis

Reviewing Editor: Michael Michaelides, NIDA-NIH Decisions are customarily a result of the Reviewing Editor and the peer reviewers coming together and discussing their recommendations until a consensus is reached. When revisions are invited, a fact-based synthesis statement explaining their decision and outlining what is needed to prepare a revision will be listed below. The following reviewer(s) agreed to reveal their identity: Umair Hassan, Simone Russo.

Reviewer 1

Authors described how 1Hz rTMS on motor cortex relates to the 2 most common electrophysiological outcomes (motor evoked potentials & TMS evoked EEG potentials). The study is very timely, needed and therefore I recommend its publication in this form or further refined form. I believe authors can revise their manuscript before final print to enhance the impact of their scientific work. The study focuses on the motor cortex but doesn't delve into the specific types of neurons (e.g., pyramidal neurons, interneurons) that might be affected by rTMS. Understanding the cellular basis could provide more nuanced insights into the observed effects. The authors mentions the induction of neuroplasticity but does not discuss the underlying mechanisms. Neuroplasticity is a complex process involving synaptic strength, dendritic branching, and even neurogenesis. A more detailed discussion would strengthen the manuscript. While the study aims to understand the temporal dynamics of rTMS effects, it doesn't discuss how these temporal patterns might relate to known neural oscillations or rhythms, which are fundamental to cortical activity and motor function. This research work observes inter-individual differences but doesn't tie these back to known sources of neural variability, such as genetic factors, that could affect neurotransmitter levels and receptor densities, thereby influencing rTMS responsiveness. Furthermore, the study does not differentiate between the effects of rTMS on inhibitory vs. excitatory neurons in the motor cortex, which could have different implications for motor control and learning. Authors briefly mentions that rTMS effects could be state-dependent but does not explore this in the context of current theories about state-dependent neural computation and information processing in the motor cortex. While the study advocates for a dose-response consideration in rTMS protocols, it doesn't discuss this in the context of known dose-response curves for neurotransmitter release or receptor activation in neural tissue. Authors focuses on the motor cortex but does not discuss how the observed changes in cortical excitability might translate to changes in motor function or behavior, which is ultimately the output of the motor cortex. The manuscript could have described how rTMS might affect synaptic efficacy, given that changes in MEPs and TEPs could be indicative of changes at the synaptic level, because the motor cortex does not operate in isolation but is part of a broader neural circuit involving other cortical and subcortical areas. The study does not discuss how rTMS of the motor cortex might affect these interconnected regions. Finally, the study aims to validate "online" rTMS-EEG and -EMG analyses but does not discuss the challenges and limitations of real-time neural monitoring, such as signal-to-noise ratio issues, which are crucial in neuroscience research. It would be better to treat these as post-hoc sorted online effects.

My comments:

Small Sample Size: The study uses only 10 healthy human participants, which may not be sufficient to generalize the findings. Were there are power calculations done for this many subject numbers? In case there were only 10 subjects, per subject and per block effects (within subject) would be clearly observed for a study to be published in an esteemed journal such as eNeuro. The limitation of comprising of lower sampling rate should be atleast mentioned very clearly in the abstract if not addressed in the revision.

Lack of Control/Sham: There is no mention of a control site/sham stimulation, which would be essential for establishing causality. I believe, by now after Conde et al 2019, we do have a consensus that control and sham conditions are quite important before drawing any conclusions in TMS-EEG work.

Healthy Participants: The study focuses on healthy individuals, which may not translate directly to clinical populations where rTMS is often used. We often forget this point and generalize our findings to everyone using rTMS - it would be quite important to clarify this either in the abstracts or better in the title!

The study finds different results compared to previous research (e.g., Helfrich et al., 2012) but attributes this to differences in the sample. This raises questions about the generalizability and comparability of these studies. The study mentions that rTMS effects may be state-dependent but does not elaborate on how this could have influenced their results or how it should be accounted for in future research. Furthermore, the study also discusses various TEP components but does not provide a clear theoretical framework for interpreting these components. This makes it difficult to understand the broader implications of the findings.

While the study acknowledges the limitation of potential shifts in coil positioning, it does not discuss how this could have affected the results, especially given that TMS effects are highly spatially specific. Were the neuronavigation event markers saved along with the EEG data? There could be one nice figure showing stability of navigational targets and if the changes in the navigation spatial targets have correlations with the effects found between the TMS, EEG and EMG? If this is not possible, this would be a limitation of the study.

Temporal Changes: The study focuses on temporal changes but does not specify the time frame clearly in which these changes occur. They do talk about number of trials (1Hz and trials can be translated into time course, but I think discussing time frames explicitly could enhance the value proposed by the work)

Global Neuronal Activity: The study uses global mean field power as a measure but does not clarify why this specific measure was chosen over others. What are the effects with other measures of cortical excitability (could be supplementary also)

Comparison with Previous Studies: The study could benefit from a more detailed comparison with existing literature, especially studies that have conflicting findings. There are some concurrent TMS-fMRI studies (online and offline both) at 1 Hz stimulation frequencies at M1 have been done in humans - I think a through literature, and concluded effect of TMS on M1 could help contextualizing these results in a much better way. I am surprise to see that N15 or any other earlier latency do not show any stronger correlation MEP. There has been concurrent TMS-fMRI work showing that suprathreshold TMS does modulates BOLD response measured using fMRI in humans so why not the TEPs? I would suggest to thoroughly check Bergmann et al TMS-fMRI review and connect your findings with the concurrent TMS-fMRI work, it is possible that BOLD does gets modulates because that's happened at much later time latency, than the TEPs and it's fine that if the conclusion in the end comes out to be that BOLD and TEPs are not correlatable, but would be worth it to include this into discussion and revise manuscript accordingly.

Artifact Rejection: The study mentions that 10-20% of EEG trials were rejected during preprocessing, which could introduce bias. On the similar note re. statistical significance - the study sets statistical significance levels at 5% for all analyses but does not mention any correction for multiple comparisons, which could increase the risk of Type I errors.

Choice of Electrodes: The study identifies specific electrodes for analysis but does not provide a rationale for why these were chosen and similarly while the study uses growth model fitting, it's not clear how well this model fits the data or if other models were considered.

While the study mentions the use of R and Matlab, it does not specify whether the code will be made available for verification or replication. I can see value for this data and analysis scripts to be publicly available.

Different TEP components and EMG data show different trends (linear, quadratic, cubic), which makes the results difficult to interpret collectively - I believe authors can present a schematic diagram framing the theory behind these trends since the study presents a lot of data but does not seem to tie it back to any existing theories or models, which could help in better understanding the implications of the findings.

The study reports average RMT and stimulation intensity but does not discuss how variations in these measures might have affected the results within the subject, and between subjects. This could be one of the limitation of the work, at least acknowledging this would be required.

The study mentions "significant but negligible correlations" between EEG and EMG data, which raises questions about the practical significance of these correlations. Also, while the single-subject analysis is interesting, it's not clear how these individual trends contribute to the overall understanding of the phenomenon under study.

Sample Size: The study acknowledges a small sample size but does not discuss how this limitation might affect the generalizability of the findings. Small sample sizes can lead to inflated Type I or Type II errors.

The study mentions that 20% of the data was eliminated due to artifacts. This is a significant proportion and could introduce bias or affect the reliability of the results. I think this once again warrants the need for open data and scripts so that they can be reproduced by at least some reviewer.

Reviewer 2

In this work the authors explore the effect of rTMS over the primary motor cortex by measuring the modulation of TMS evoked EEG and EMG responses over time.

They investigate the EEG and EMG responses evoked by TMS in a cohort of 10 participants (6 F). To account for TEPs variability between subjects, the authors put a valuable effort in providing single subject analyses.

The title reflects the topic of the paper. It could be beneficial to explicit in the title that the employed electrophysiological measures were EEG and EMG.

The introduction and the discussion are clear, while the results section is somehow not intuitive.

While this study clearly demonstrates the feasibility of EEG monitoring during rTMS, the scientific message provided in the results is not very clear.

What is the meaning of fitting a quadratic trend? Should we expect an increase, a decrease, or an oscillation of the EEG response to rTMS over time? And what is its biological meaning?

The authors show a negligible correlation between MEP and EEG responses, in line with multiple rTMS studies. It would be worth discussing this difference with respect to Fecchio et al (Fecchio, PLOS, 2017), as this may provide intriguing hypothesis on the plasticity mechanisms. This topic is particularly relevant since the authors localize N100 in the somatosensory cortex.

The authors report an increase in the amplitude of the TMS response over time. A potential risk of these experiments is that drowsiness may emerge over time and result in larger TEP responses, especially in the early components (Massimini, Science, 2005). It would be important to report whether the experimenters applied specific techniques to control for it or disclose the emergence of drowsiness as a potential limitation.

The authors write "Please note that RMT is higher in EEG measurements due to higher cortex-coil distance". Is this due to the presence of the EEG cap? If so, I suggest the authors to explicitly write it, as it can be not intuitive for a non-expert reader.

The authors focus on the MEP evoked in FDI. Yet, achiving such high specificity with MEPs is challenging and the studied MEPs are likely to engage other muscles. It is important to explicit this limitation of the study, as it allows for a more careful interpretation of the MEP and MEP-EEG results.

In the abstract (line 12) there is a typo: "the objective OF this study"

Line 289-290 is not clear, probably due to a typo.

Line 306: there is a typo "á".
